# Inverted ILM Flap Technique in Idiopathic Full-Thickness Macular Hole Surgery: Functional Outcomes and Their Correlation with Morphologic Findings

**DOI:** 10.1155/2021/6624904

**Published:** 2021-02-11

**Authors:** Paolo Carpineto, Enrico Borrelli, Luca Cerino, Daniele Guarini, Agbeanda Aharrh-Gnama, Vincenzo Ciciarelli, Carla Iafigliola, Leonardo Mastropasqua

**Affiliations:** ^**1**^ Ophthalmology Clinic, Department of Medicine and Science of Ageing, University G. D'Annunzio Chieti-Pescara, Chieti, Italy; ^**2**^ Medical Retina & Imaging Unit, Department of Ophthalmology, University Vita Salute, IRCCS Ospedale San Raffaele, Milano, Italy

## Abstract

**Objectives:**

The inverted internal limiting membrane (ILM) flap technique has been shown to increase the success rate in large full-thickness macular holes (FTMHs) and in FTMHs associated with high myopia. The aim of our study was to confirm the efficacy and safety of inverted ILM flap technique in idiopathic FTMHs independent of their dimensions and to assess functional outcomes and their correlation to morphologic findings.

**Methods:**

Sixteen consecutive patients affected by idiopathic FTMH were enrolled in this prospective study. The preoperative mean (±SD) diameter of the FTMH was 422 (±106) *µ*m. All patients underwent vitrectomy and ILM peeling with inverted ILM flap. At 1-, 3-, and 6-month postoperative visits, visual acuity measurement, indirect ophthalmoscopy, and microperimetry were performed, and the foveal contour and the integrity of the ellipsoid zone (EZ) and external limiting membrane (ELM) were investigated using spectral domain optical coherence tomography (SD-OCT).

**Results:**

At six months postoperatively, 15 out of 16 (93.75%) patients obtained FTMH closure. The mean best corrected visual acuity (BCVA) improved from 1.1 LogMAR to 0.3 LogMAR, and the mean retinal sensitivity (MS) improved from 7.2 to 23.4 dB. ELM defects were evident in 1 out of 16 (6.25%) eyes, and EZ defects were detected in 2 out of 16 (12,50%) eyes. A statistically significant relationship was observed between BCVA, MS, and EZ reconstitution at each follow-up visit.

**Conclusions:**

Results confirm that the inverted ILM flap technique is a safe and effective option for FTMH treatment and show a strong correlation between higher BCVAs and MSs and EZ reconstitution after surgery.

## 1. Introduction

Idiopathic full-thickness macular hole (FTMH) is a sight-threatening vitreoretinal disorder, which affects approximately 8.7 eyes per 100,000 per year [1, 2]. Surgery for FTMH has undergone important advancements since Kelly and Wendel [3] first described pars plana vitrectomy (PPV) to treat FTMH. Before the introduction of PPV, the spontaneous closure rate for FTMH was estimated to be approximately 4% [4]. The introduction of PPV, thus, increased the closure rate to 58% [3]. Nowadays, given the improvements in diagnostic and surgical techniques and instrumentation, the closure rate increased to as high as 90% [5] and the additional use of internal limiting membrane (ILM) peeling represented a fundamental progression in FTMH surgery [6]. Recently, the inverted ILM flap was introduced as an alternative surgical technique, and it was shown to increase the success rate in large FTMHs, in FTMHs associated with high myopia, and in refractory FTMHs [7–11].

Some studies recently demonstrated that the intraoperative optical coherence tomography (iOCT) is a useful tool to guide intraoperative decision in the attempt to improve both the morphological and functional outcome in FTMH surgery [12–14].

Here, we report sixteen cases of patients who underwent PPV with the ILM inverted-flap technique for FTMH. iOCT was used to assess the correct ILM flap placement. Postoperatively functional results as best corrected visual acuity (BCVA) and mean retinal sensitivity (MS) were correlated to spectral domain optical coherence tomography (SD-OCT) morphologic findings as external limiting membrane (ELM) and ellipsoid zone (EZ) integrity.

## 2. Materials and Methods

In this prospective, interventional study, 16 eyes from 16 consecutive patients (10 males, 6 females) suffering from idiopathic FTMH were enrolled, independently from FTMH diameter. All of them subscribed written informed consent prior to inclusion.

The study protocol was approved by the local ethics committee and was accordant to the principles of the Declaration of Helsinki.

Exclusion criteria were any history of previous vitreoretinal surgery and any other vascular or degenerative retinal pathology (i.e., diabetic retinopathy, hypertensive retinopathy, retinal vein/artery occlusion, high myopia, and macular degeneration).

All patients underwent a global ophthalmic evaluation including BCVA measurement, anterior segment examination, and dilated fundoscopy. All of them were imaged using SD-OCT (Cirrus HD-OCT, Carl Zeiss Meditec, Inc., Dublin, CA). A HD cross-imaging protocol consisting of 10 scans, 5 horizontal and 5 vertical (8x averaged), centered on the fovea was adopted. To define FTMHs size, the horizontal diameter at the narrowest point was measured, according to FTMH classification of the International Vitreomacular Traction Study (IVTS) group [15]. In addition, microperimetry (MP-3, Microperimetry-3, Nidek, Japan) was performed to evaluate MS in a 2-degree-wide approximately circular area centered on the fovea before surgery.

One surgeon (PC) performed all the interventions. All eyes underwent PPV with a 25-gauge transconjunctival system (Constellation^®^, Alcon Inc, Fort Worth, Texas, USA). Core vitrectomy and MembraneBlue-Dual^®^ (Dutch Ophthalmics Research Centre, Zuidland, The Netherlands) staining were performed. A contact lens (Alcon, Geneva, Switzerland) was placed for macular surgery. If present, an epiretinal membrane (ERM) peeling was first performed. Next, ILM was peeled in an area of about two optic disc diameters all around the FTMH. During this maneuver, ILM was left attached to the edge of the FTMH. Then, the flap was reduced by vitrectomy and gently folded and filled into the FTMH (fold and fill technique). At the end of surgery, the vitreous cavity was filled with an unexpandable mixture of air and sulfur hexafluoride (SF_6_). The iOCT system (CALLISTO eye®, Carl Zeiss Meditec Inc., Dublin, CA, USA) was used during the surgery to assess the ILM peeling and to confirm the correct ILM flap placement, even after the air-fluid exchange. Postoperatively, patients were asked to adopt a face down posture for 3 days.

Patients were reexamined at 1 week and 1, 3, and 6 months postoperatively, and at each visit, they underwent comprehensive eye examination including BCVA measurement, anterior and posterior segment examination, microperimetry, and SD-OCT imaging. During each postoperative visit, the foveal contour and the presence of EZ and ELM defects were investigated.

Postoperative foveal contour was characterized according to its cross-sectional morphology on SD-OCT into the following categories: U-shaped, V-shaped, and W-shaped (irregular). The W-shaped contour was attributed to persistent FTMHs.

## 3. Statistical Analysis

Statistical analysis was performed using MedCalc software (version 19.3.1, MedCalc Software Ltd., Ostend, Belgium). To detect departures from normal distribution, the D'Agostino–Pearson test was performed for all variables. The D'Agostino–Pearson test (Sheskin, 2004) computes a single *p*-value for the combination of the coefficients of skewness and kurtosis.

The variables were presented as follows: dichotomous variables as status of FTMH after surgery (i.e., open/closed) and reconstitution of EZ; continuous variables as FTMH size, MS, and BCVA. Since W-shaped foveal contour was attributed to persistent FTMHs, foveal profile was treated as a dichotomous variable (U-shaped/V-shaped).

In order to test the influence of age, preoperative FTMH size, and preoperative BCVA on BCVA and MS at the postsurgical month 6 visit, the multivariate regression model was applied.

A correlation analysis was used to relate BCVA and MS to each other before and after surgery.

Moreover, a logistic regression analysis was performed to test the relationship between EZ reconstitution and both BCVA and MS at the postsurgical 1-, 3-, and 6-month visits. In addition, logistic regression analysis was also used to relate EZ reconstitution after surgery and preoperative FTMH size.

Finally, foveal contour was related to both BCVA and MS at the postsurgical 1-, 3-, and 6-month visits through a logistic regression analysis.

The chosen level of statistical significance was a *p* value< 0.05.

## 4. Results

The mean ± SD age was 69.4 ± 5.5 years (range: 59.0–78.0 years). All eyes were pseudophakic, and their axial length was lower than 26 mm. All patients were affected by idiopathic FTMH. At preoperative SD-OCT examination, the mean (±SD) diameter of the FTMH was 422 (±106) µm (range: 110–589 *µ*m). Microperimetry performed before surgery resulted in an MS of 7.16 ± 5.64 dB (mean ± SD) (range: 1.6–16.1 dB). Furthermore, preoperative patients' characteristics are shown in Table 1.

During surgery, the correct positioning of the ILM inside the FTMH was checked in all patients using iOCT.

A complete closure of the FTMH was achieved in 15 out of 16 eyes (93.75%) at each postoperative visit (Table 2).

At SD-OCT examinations, the postoperative foveal contour was stable throughout the follow-up (Table 2), while ELM defects were displayed in 4 out of 16 (25%) eyes at the 1-week visit and in 1 out of 16 (6.25%) eyes at 1-, 3-, and 6-month visits. Discontinuity of the EZ band was noted in all 16 eyes at 1-week examination, in 9 out of 16 (56.25%) eyes at the 1-month visit, in 7 out of 16 (43.75%) eyes at the 3-month visit, and in 2 out of 16 (12,50%) eyes at the 6-month visit (Table 3).

**Figure 1:**
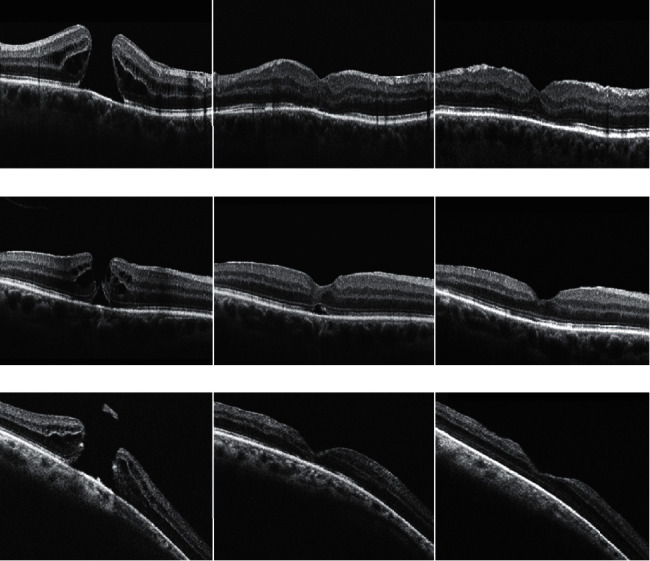
Baseline (left), 1-month (middle), and 6-month (right) follow-up SD-OCT images from three patients (a), (b), and (c) with FTMH and undergoing the inverted ILM flap technique. At the 6-month follow-up visit, the EZ reconstitution was evident.

Furthermore, all patients displayed the presence of a hyperreflective material at the fovea, which is secondary to the ILM flap filled into the FTMH (shown in Figure 1).

The mean (±SD) BCVA was 1.1 ± 0.4 LogMAR at baseline and 0.3 ± 0.4 LogMAR at the 6-month follow-up visit. In detail, 15 out of 16 eyes had an improvement of the visual acuity after surgery (93%), while 1 eye experienced a reduction in BCVA after surgery.

MS in a 2-degree-wide area centered on the fovea measured with microperimetry was 23.4 ± 6.4 dB (mean ± SD) at the 6-month postoperative visit. In details, after surgery, MS improved in 15 out of 16 eyes (93%), while worsening in 1 eye.

No statistically significant relationship was observed between BCVA at the 6-month follow-up visit and preoperative BCVA (*p*=0.12), age (*p*=0.6), FTMH minimum (*p*=0.4), and base (*p*=0.7) diameter.

Similarly, the MS measured at the 6-month follow-up visit was not significantly related to preoperative BCVA (*p*=0.35), age (*p*=0.76), FTMH minimum (*p*=0.85), and base (*p*=0.92) diameter.

On the contrary, a significant association was observed between preoperative BCVA and MS (*p* < 0.0001), as well as between postoperative BCVA and MS at 1- (*p* < 0.0001), 3- (*p* < 0.0001), and 6-month (*p* < 0.0001) follow-up visits.

A statistically significant relationship was also observed between BCVA and EZ reconstitution evaluated at 1-month (*p*=0.01), 3-month (*p*=0.001), and 6-month (*p*=0.0005) follow-up visits, with a linear relationship between the mean BCVA and the percentage of eyes with the EZ reconstitution at each visit (*p*=0.02).

Also, MS was significantly related to EZ reconstitution evaluated at 1- (*p*=0.0007), 3- (*p*=0.0001), and 6-month (*p*=0.0005) follow-up visits, with a linear relationship between the mean MS and the percentage of eyes with the EZ reconstitution at each visit (*p*=0.02).

Finally, foveal contour evaluated at each postoperative visit had no statistically significant relationship with BCVA (*p*=0.92, 0.99, and 0.35 at 1-, 3-, and 6-month postoperative visit, respectively) and MS (*p*=0.74, 0.83, and 0.26 at 1-, 3-, and 6-month postoperative visit, respectively).

## 5. Discussion

In the last decade, several improvements to the FTMH surgery have been proposed to meliorate both anatomical and functional outcomes. ILM peeling was demonstrated to be an important surgical step in FTMH surgery, since it relieves the tangential tractional forces occurring around the fovea and ensures removal of the whole epiretinal tissue [16–18]. The ILM peeling was demonstrated to significantly increase the anatomic success rate in FTMH surgery, while no differences in visual acuity were observed between patients with and without ILM peeling [6, 19].

Several surgical techniques have been proposed in order to optimize surgical outcomes, considering that ILM peeling itself induces significant changes in the retinal structure [20].

In 2010, Michalewska and colleagues [7] described the inverted ILM flap technique. It consists in leaving a fragment of ILM attached to the margins of the FTMH. The ILM flap is, thus, inverted upside down and filled inside the FTMH. Some important studies have proved that the inverted ILM flap technique improves both the rate of FTMH closure and the visual outcome.

Recently, Rizzo et al. [21] performed a comparative study of patients affected by idiopathic or myopic FTMH and undergoing PPV and ILM peeling with and without the ILM flap technique. The authors demonstrated that the ILM flap technique is a safe and effective surgical step. Moreover, they displayed an increased FTMH closure rate (95.6% vs. 78.6%) and visual acuity improvement in eyes with large FTMHs undergoing the ILM flap technique. On the contrary, small FTMHs (<400 micron) did not experience a significant improvement in anatomical and functional outcomes using the ILM flap technique.

Rossi et al. [9] compared two different inverted ILM surgical techniques, the cover one and the fill one, and found similar closure rates and postoperative visual acuity at 3 months. However, the fill technique was found to close better larger holes. On the other hand, Faria et al. [22] recently showed that the fill technique is associated with poorer anatomical and visual results compared with ILM placed over the hole.

Also, Ghassemi et al. [23] compared three different techniques of inverted ILM flap in large idiopathic FTMH surgery, hemicircular ILM peeling with temporal-inverted flap (group A), circular ILM peeling with temporal-inverted flap (group B), and superior inverted-flap technique (group C), and concluded that all the techniques have comparable, high, and effective results.

The inverted ILM flap technique is not without intraoperative complications and technical difficulties. In fact, the inverted ILM flap may detach spontaneously during the air-fluid exchange [7].

Some authors have proposed modified techniques to overcome this complication [24, 25]. Andrew et al. [25] described a technique consisting in using a viscoelastic cap in order to ensure retention of the ILM flap within the hole. Conversely, Shin and colleagues [26] used perfluorocarbon to reduce the ILM flap movement before the air-fluid exchange. Additional surgical manipulation steps of the ILM flap (e.g., ILM trimmed, ILM tuck inside the hole, and ILM massage) have been described and proposed. However, these additional steps were demonstrated not to improve the postsurgical outcome [24].

In a recent paper, we presented three cases of idiopathic FTMH treated with PPV followed by ILM peeling and the “fold and fill” technique [14]. The use of iOCT during surgery resulted important for several reasons, including (i) the identification of subclinical residual membranes and ILM, whose presence requires additional peeling, (ii) the simplification of the procedure of flap inverting, and (iii) the confirmation of the position of the inverted ILM flap following complete air-fluid exchange, which may generate turbulences in the vitreous chamber.

In this study, we reported structural and functional outcomes of 16 idiopathic FTMH eyes undergoing inverted the ILM flap technique assisted by iOCT. Our study confirmed that this technique may be considered as a safe and effective surgical option for idiopathic FTMHs, improving both functional and anatomic outcomes, independently from FTMH dimension. In detail, we showed that this technique is associated with a high rate of postsurgical closure, assuming that 93.75% of our enrolled eyes experienced successful FTMH closure and that other authors reported that the ILM peeling surgery without ILM flap technique has an estimated rate closure of 78% [17]. Therefore, it has been supposed that Müller cell fragments contained in the ILM flap may induce glial cell proliferation, which fills the FTMH and, thus, supports FTMH closure [5].

Moreover, we noted an overall improvement in visual acuity after surgery (93.75% in our case series versus 70% in the previous case series without ILM flap [13–15]), which may be related to an increased restoration of foveal photoreceptors.

Some doubts have been raised that the ILM flap, filling the hole, might prevent visual acuity recovery interfering with photoreceptors reconstitution and acting as a plug of fibrotic scar tissue [18, 27]. But, the ILM tissue is known to work rather as a scaffold for tissue proliferation, promoting photoreceptors restoration and providing them a guidance for a proper positioning [5]. In addition, as stated by Rossi et al. [9], using the fill technique, the ILM acts as a filler, glue, and scaffold at the same time. The FTMH closes early in the postoperative period, and no outer retinal cysts are visible at 1 or 3 months.

Bottoni et al. [28] described the progressive restoration of the outer retinal layers after vitrectomy and ILM peeling for idiopathic FTMH. While ELM needed 1–3 months to achieve complete reconstitution, up to 12 months could be necessary for EZ restoration. The authors observed that the progressive repair of ELM, EZ, and the outer nuclear layer (ONL) was strongly related to visual acuity improvement and both structural and functional restoration occurred months after the FTMH closure.

More recently, Morawski et al. [29] demonstrated that the EZ recovery is the structural feature most highly related with BCVA improvement after the inverted ILM flap technique for FTMH treatment.

In their recent research article, Mete et al. [30] observed no difference between the inverted-flap group and the ILM peeling group regarding timing and restoration rate of the ELM and EZ, affirming that the presence of the ILM plug does not prevent the physiological healing process occurring after ILM peeling [18].

Our series demonstrated an early and increased restoration of the ELM, while the restoration of the EZ occurred later. However, a statistically significant association between EZ integrity and both BCVA and microperimetric MS was evident at 1-, 3-, and 6-month evaluation. Indeed, the clearest improvement of each eye visual function was associated with EZ restoration at SD-OCT imaging and lower BCVAs (0.6 LogMAR and 2 LogMAR), and MSs (18.9 dB and 1.2 dB) were observed at the 6-month visit in the two eyes with persisting EZ discontinuity.

The apparent disagreement between ELM and EZ restoration may be explained by a lower function of the photoreceptors after surgical injury. Indeed, the EZ is called so because its reflective signal has been suggested to originate from the photoreceptor inner segment ellipsoids, which contain a high density of mitochondria [31]. Thus, the EZ reflectivity may be considered as a detector of the photoreceptors function, rather than a morphological feature of the outer retinal layers. In agreement with the latter aspect, several important studies demonstrated that the macular function gradually improves after FTMH closure [32, 33].

## 6. Conclusions

This case series confirmed that the ILM flap technique in its fold and fill variant is a safe and effective surgical option for idiopathic FTMH independent of hole size. iOCT is a useful tool in demonstrating ILM flap integrity and its correct positioning during and after air-fluid exchange. The ILM tissue filled inside the hole may improve both morphological and functional outcomes. Retinal sensitivity measured with MP-3 is directly related to visual acuity. EZ restoration after surgery is the structural OCT finding mostly related to BCVA and MS improvements. Future longitudinal case-control studies may shed further light on these aspects.

## Figures and Tables

**Table 1:** Mean preoperative patients' characteristics.

Age ± S.D. (years)	Male/female	BCVA ± S.D. (logMAR)	Minimum FTMH diameter ± S.D. (*µ*m)	Base FTMH diameter ± S.D. (*µ*m)	MS ± S.D. (Decibel)
69.125 ± 5.35	10/6	1.137 ± 0.44	422 ± 106	1124 ± 216	7.16 ± 5.64

**Table 2:** Postoperative foveal appearance at SD-OCT examination.

	1 week	1 month	3 months	6 months
Closed/open	15/1	15/1	15/1	15/1
U-shaped	10	9	9	9
V-shaped	5	6	6	6
W-shaped	1	1	1	1

**Table 3:** Outer retinal layer characteristics at different follow-ups.

	1 week	1 month	3 months	6 months
ELM defects (%)	4/16 (25%)	1/16 (6.25%)	1/16 (6.25%)	1/16 (6.25%)
EZ defects (%)	16/16 (100%)	9/16 (56,25%)	7/16 (43,75%)	2/16 (12,5%)

## Data Availability

All data generated or analyzed during the current study are available from the corresponding author on reasonable request.
